# Investigating the origin of insect metamorphosis

**DOI:** 10.7554/eLife.94410

**Published:** 2023-12-21

**Authors:** Xavier Belles

**Affiliations:** 1 Evolution of Insect Metamorphosis Lab, Institute of Evolutionary Biology, CSIC-Pompeu Fabra University Barcelona Spain

**Keywords:** evolution, metamorphosis, development, insects, firebrat, juvenile hormone

## Abstract

Experiments exploring the role of juvenile hormone during the life cycle of firebrat insects provide clues about the evolution of metamorphosis.

**Related research article** Truman JW, Riddiford LM, Konopová B, Nouzova M, Noriega F, Herko M. 2023. The embryonic role of juvenile hormone in the firebrat, *Thermobia domestica*, reveals its function before its involvement in metamorphosis. *eLife* **12**:RP92643. doi: 10.7554/eLife.92643.

Given Darwin’s longstanding interest in entomology, it is possible he had insects and metamorphosis in mind when he wrote about “endless forms most beautiful” in the final sentence of *On the Origin of Species*. More than half of the 2.2 million animal species that have been described are insects, and 98% of these undergo some form of metamorphosis during their lives.

Insects have three basic types of life cycle: ametaboly (no metamorphosis), hemimetaboly (gradual metamorphosis), and holometaboly (complete metamorphosis). To begin with, all insects were ametabolous, and evolved the ability to mature first via gradual metamorphosis and later via complete metamorphosis (Belles, 2020). Although most insects are holometabolous, this life cycle could not have evolved without hemimetaboly emerging first. However, we do not fully understand how ametabolous insects evolved to become hemimetabolous.

Ametabolous insects are wingless and hatch from their eggs as a miniature version of their adult form. The resulting young, known as nymphs, do not undergo any dramatic physical changes, but successively molt their exoskeleton to produce a series of larger, more mature versions known as instars. Hemimetabolous insects, like cockroaches, also hatch as nymphs that resemble the adult but lack fully developed wings and genitalia. This group of insects molt as they grow until they reach a last nymphal instar, at the end of which they shed their exoskeleton for the last time whilst undergoing metamorphic changes that produce their mature genitalia and wings.

The addition of metamorphosis to the life cycle likely arose due to the emergence of wings during evolution. While wings were a successful innovation, molting with wings is mechanically complicated – only mayflies, a small order of insects with about 3,000 extant species still do it. Therefore, ceasing to molt after forming adult wings was the next step that completed the invention of hemimetaboly (Belles, 2019). However, it is not known which features of ametabolous insects contributed to the evolution of metamorphosis. Now, in eLife, James Truman from the University of Washington and co-workers report that changing levels of a chemical signal known as juvenile hormone may have had a significant role (Truman et al., 2023).

Juvenile hormone is an important player in post-embryonic development, as it represses metamorphosis in both hemimetabolous and holometabolous insects until they reach a critical size to transform into reproductively competent adults. Truman et al. – who are based at institutes in the United States and the Czech Republic – found that juvenile hormone is present in the last quarter of embryogenesis in the ametobolous firebrat insect *Thermobia domestica* (upper panel in Figure 1). Treatments suppressing the hormone impaired the differentiation and maturation of embryo structures, as well as hatching. These defects were corrected when juvenile hormone was re-introduced, validating the hormone suppression experiments.

**Figure 1.**
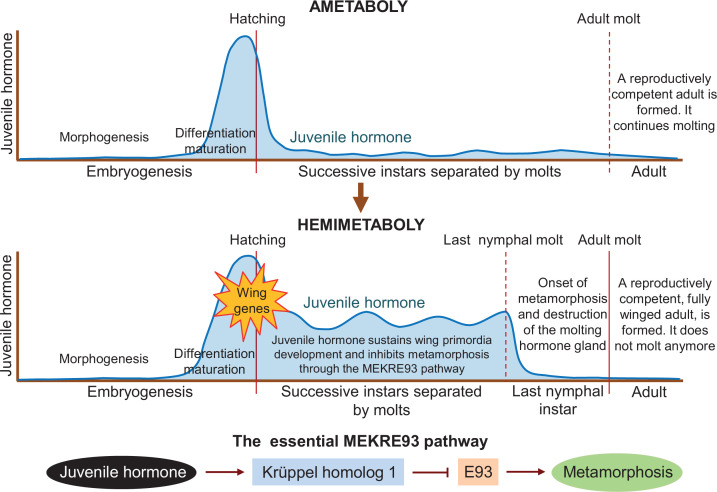
Differences in juvenile hormone levels during the life cycle of ametabolous and hemimetabolous insects. During embryogenesis in ametabolous insects (upper panel), the level of juvenile hormone (dark blue line) remains low during morphogenesis, and then increases rapidly after the shape of the embryo has been established, resulting in the onset of differentiation and maturation. Once the nymph hatches from the egg, the hormone level falls and remains low as the nymph successively sheds its exoskeleton to accommodate its growth. After the adult molt, a fully-grown reproductively competent adult emerges, which continues to molt throughout its lifetime. In hemimetabolous insects (middle panel), the level of juvenile hormone follows a similar pattern during embryogenesis. After hatching, however, it remains relatively high as the nymph molts. Juvenile hormone sustains the development of early structures of the wing (known as primordia) and inhibits metamorphosis via the MEKRE93 pathway. In this pathway, juvenile hormone induces the production of Krüppel homolog 1 which, in turn, represses another protein called E93 (bottom panel). When juvenile hormone declines in the last nymphal instar, this leads to a rise in E93 which triggers the insect to undergo metamorphosis as it molts into its final adult form (red line). E93 also promotes the destruction of the gland responsible for producing the molting hormone, preventing the insect from molting again after becoming an adult.

The first three quarters of embryogenesis, when there is no juvenile hormone, are characterized by morphogenetic processes that determine the shape of the organism. Differentiation and maturation processes then take over in the last quarter when juvenile hormone is present. Secretion of juvenile hormone therefore appears to mark the end of morphogenesis and the onset of differentiation and maturation of tissues and organs in the firebrat embryo. Artificially applying juvenile hormone during the earlier stages of embryogenesis triggered differentiation processes, providing further evidence for this shift.

Truman et al. report that hemimetabolous insects display a similar pattern of juvenile hormone secretion during embryogenesis. However, juvenile hormone levels during the nymphal period are low in the firebrat but high in hemimetabolous insects. This led the team to suggest that the role of juvenile hormone in the embryo – promoting differentiation while repressing morphogenetic changes – may have been extended into post-embryonic development in the ancestral insects from which the first hemimetabolans originated. This event would concur with the activation of the genes responsible for forming the early structures of the wing (middle panel in Figure 1).

Results from previous studies indicate that continuous juvenile hormone secretion has two important roles in the nymphs of hemimetabolous insects. One is to promote wing development by maintaining the production of the protein factor Broad-complex (Erezyilmaz et al., 2006). The other is to prevent metamorphosis by inducing the production of the protein Krüppel homolog 1 (Konopova et al., 2011; Lozano and Belles, 2011). Importantly, Krüppel homolog 1 represses the production of another protein called E93 (Belles and Santos, 2014), which is the trigger of metamorphosis (Ureña et al., 2014). This pathway, known as MEKRE93, is the essential axis that regulates metamorphosis in all insects (lower panel in Figure 1). The protein E93 also causes the gland that secretes the molting hormone to be destroyed after the last nymphal instar, preventing the adult from molting again (Kamsoi and Belles, 2020).

The extant firebrat also produces Broad-complex, Krüppel homolog 1, and E93, as did probably ancestral ametabolous insects (Fernandez-Nicolas et al., 2023; Truman et al., 2023). This suggests that these proteins just needed to take on new functions in order for metamorphosis to evolve. In the future, it would be interesting to study the role of these proteins in firebrat insects, and the molecular mechanisms that allowed them to change their function. Although not an easy task, this would provide important insights in to how metamorphosis evolved.
